# Non-alcoholic fatty liver disease and cerebral small vessel disease in Korean cognitively normal individuals

**DOI:** 10.1038/s41598-018-38357-x

**Published:** 2019-02-12

**Authors:** Hyemin Jang, Danbee Kang, Yoosoo Chang, Yeshin Kim, Jin San Lee, Ko Woon Kim, Young Kyoung Jang, Hee Jin Kim, Duk L. Na, Hee Young Shin, Mira Kang, Eliseo Guallar, Juhee Cho, Sang Won Seo

**Affiliations:** 1 Department of Neurology, Samsung Medical Center, Sungkyunkwan University School of Medicine, Seoul, 06351 Korea; 2 Neuroscience Center, Samsung Medical Center, 06351 Seoul, Korea; 3 Center for Clinical Epidemiology, Samsung Medical Center, 06351 Seoul, Korea; 4 Health Promotion Center, Samsung Medical Center, 06351 Seoul, Korea; 5 Department of Occupational and Environmental Medicine, Kangbuk Samsung Hospital, Sungkyunkwan University, School of Medicine, Seoul, Korea; 6 Department of Neurology, Kyung Hee University Hospital, Seoul, Korea; 7 Department of Neurology, Chonbuk National University Hospital, Chonbuk National University Medical School, Jeonju, Korea; 8 Department of Health Sciences and Technology, Sungkyunkwan University, Seoul, 06351 Korea; 9 Clinical Research Design and Evaluation, SAIHST, Sungkyunkwan University, Seoul, 06351 Korea; 10 Department of Epidemiology, Johns Hopkins Medical Institutions, Baltimore, USA; 11 Department of Medicine, Johns Hopkins Medical Institutions, Baltimore, USA; 12 Welch Center for Prevention, Epidemiology and Clinical Research, Johns Hopkins Medical Institutions, Baltimore, USA

**Keywords:** Non-alcoholic fatty liver disease, Cerebrovascular disorders

## Abstract

We aimed to investigate the association between nonalcoholic fatty liver disease (NAFLD) and cerebral small vessel disease (CSVD) burden, especially according to the NAFLD severity. A total of 1,260 participants were included. The CSVD burden was assessed with white matter hyperintensities (WMH), lacunes, and microbleeds (MBs) on brain MRI. An ultrasound diagnosis of fatty liver was made based on standard criteria, and the Fibrosis-4 (FIB-4) index was used to classify participants with NAFLD with having a high-intermediate (FIB-4 ≥1.45) or low (FIB-4 < 1.45) probability of advanced fibrosis. A multivariable logistic regression analysis was used to assess the association between NAFLD and the presence of moderate to severe WMH, lacunes, and MBs. NAFLD had a significant association only with moderate to severe WMH (OR: 1.64, 95% CI: 1.10–2.42), even after controlling for cardiometabolic risk factors. A linear trend test showed a significant association between the severity of NAFLD fibrosis and the presence of moderate to severe WMH (*p* for trend <0.001). Our findings suggest that NAFLD, especially NAFLD with fibrosis, has a significant association with the presence of moderate to severe WMH in cognitively normal individuals, and NAFLD severity predicted more frequent moderate to severe WMH.

## Introduction

Nonalcoholic fatty liver disease (NAFLD) is the most common cause of chronic liver disease, and the prevalence is rapidly increasing worldwide^1,2^. NAFLD has attracted growing attention in terms of its relation with not only hepatic complications, but also with cardiometabolic risk factors, such as hypertension, insulin resistance, and obesity^3^.

There is increasing evidence that NAFLD may affect brain health. A recent study showed that NAFLD is associated with learning and memory, as measured by symbol digit learning tests^4^. However, the pathobiology of this association remains unclear. Cerebral small vessel diseases (CSVD), such as white matter hyperintensities (WMH), lacunes, and microbleeds (MBs), are major causes of cognitive impairment. Studies have demonstrated an association between certain cardiometabolic risk factors, such as hypertension and diabetes, with CSVD development. Considering the role of NAFLD as an independent cardiometabolic risk factor^5–7^, it is reasonable to expect NAFLD to be associated with the development of CSVD. In fact, a recent study revealed that the fibrosis severity of NAFLD predicted the presence of WMH^8^. However, this study investigated a small number of study subjects, and the relationships between NAFLD and other CSVD markers, such as lacunes and MBs, which have different pathobiologies from WMH, have not been thoroughly studied.

Therefore, in this study, we aimed to investigate the association between NALFD, as assessed by ultrasonography (US), and CSVD burden, measured by magnetic resonance imaging (MRI), in a large sample of cognitively normal individuals. We hypothesized that: (1) NAFLD is associated with the presence of lacunes and MBs, as well as WMH, independent of other cardiometabolic risk factors; and (2) their significant association might depend on NAFLD severity.

## Results

### Subjects characteristics

Among 1,260 patients, 498 patients (39.6%) had NAFLD at baseline and 239 patients (19.0%) were categorized as having NAFLD with an intermediate to high FIB-4 (≥1.45) (Table 1). The demographic data of categorized patients according to NFS is shown in Table 2. Participants with NAFLD were more likely to be older, male, smokers, moderate alcohol drinkers, and to have a higher BMI and a higher frequency of hypertension and diabetes compared to those without NAFLD. In terms of CSVD markers, while only 9.6% of patients without NAFLD had moderate to severe WMH, 13.1% of patients with NAFLD with a low FIB-4 (<1.45) and 18.8% of NAFLD patients with an intermediate to high FIB-4 (≥1.45) had moderate to severeWMH. These results were statistically different. However, the prevalence of MBs and lacunes did not vary among the three groups.

**Table 1:** Study participant characteristics according to NAFLD FIB-4 score (*n* = 1,260).

Characteristic	NAFLD			*p* value
	No	Yes		
	(*n* = 762)	FIB-4 < 1.45 (*n* = 259)	FIB-4 ≥ 1.45 (*n* = 239)	
Age (years)	63.8 (7.4)	61.1 (6.4)*	66.5 (6.2)*†	<0.01
Female sex	410 (53.8)	133 (51.4)	77 (32.2)	<0.01
>middle school education	306 (40.2)	117 (45.2)	105 (43.9)	0.29
MMSE (%)	27.9 (1.8)	28.2 (1.6)	27.6 (2.1)	<0.01
Hypertension	339 (44.5)	146 (56.4)	155 (64.9)	<0.01
Diabetes	95 (12.5)	61 (23.6)	60 (25.1)	<0.01
Hyperlipidemia	233 (30.6)	96 (37.1)	91 (38.1)	0.13
BMI (kg/m2)	23.0 (2.5)	25.3 (2.8)*	25.6 (2.7)*	<0.01
Smoking				<0.01
Never	453 (59.5)	138 (53.3)	113 (47.3)	
Past or current smoker	249 (32.7)	101 (39.0)	110 (46.0)	
Unknown	60 (7.9)	20 (7.7)	16 (6.7)	
Alcohol intake				<0.01
None	494 (64.8)	163 (62.9)	121 (50.6)	
Moderate	268 (35.2)	96 (37.1)	118 (49.4)	
Systolic blood pressure (mmHg)	123.6 (18.0)	125.2 (16.7)	127.3 (18.0)*	0.02
Diastolic blood pressure (mmHg)	73.5 (10.5)	76.0 (10.4)*	76.6 (10.5)*	<0.01
Fasting glucose (mg/dL)	95.0 (14.7)	106.4 (24.9)*	104.5 (21.2)*	<0.01
HDL cholesterol (mg/dL)	58.8 (15.1)	51.2 (13.3)*	50.2 (11.7)*	<0.01
Triglycerides (mg/dL)	89 (67–121)	133 (98–184)*	129 (88–176)*	<0.01
Total cholesterol (mg/dL)	193.2 (36.3)	194.4 (37.4)	191.8 (35.6)†	0.73
CSVD markers (≥1)	165 (21.6)	61 (23.6)	81 (33.9)	<0.01
Presence of moderate to severe WMH	73 (9.6)	34 (13.1)	45 (18.8)	<0.01
Presence of microbleeds	74 (9.71)	24 (9.3)	31 (13.0)	0.30
Presence of lacunes	64 (8.4)	19 (7.3)	30 (12.7)	0.09

Data are presented as means (SDs) or numbers (percentages).

*NAFLD* nonalcoholic fatty liver disease, *FIB-4* Fibrosis-4 index, *BMI* body mass index, *HDL* high-density lipoprotein, *MMSE* Mini-Mental State Examination, *CSVD* cerebral small vessel disease, *WMH* white matter hyperintensities.

*P < 0.05 compared to no NAFLD; ^†^P < 0.05 compared to FIB-4 < 1.45.

**Table 2:** Study participant characteristics according to NAFLD NFS score (*n* = 1,260).

Characteristic	NAFLD			*p* value
	No	Yes		
	(N = 762)	NFS < −1.455 (n = 197)	NFS ≥ −1.455 (n = 301)	
Age (years)	63.8 (7.4)	61.3 (6.4)*	65.2 (6.7)*†	<0.01
Female sex	410 (53.8)	106 (53.8)	104 (34.6)	<0.01
>middle school education	306 (40.2)	89 (45.2)	133 (44.2)	0.29
MMSE (%)	27.9 (1.8)	28.2 (1.6)	27.6 (2.1)	<0.01
Hypertension	339 (44.5)	102 (51.8)	199 (66.1)	<0.01
Diabetes	95 (12.5)	19 (9.6)	102 (33.9)	<0.01
Hyperlipidemia	233 (30.6)	75 (38.1)	112 (37.2)	0.13
BMI (kg/m^2^)	23.0 (2.5)	24.4 (2.5)*	26.1 (2.7)*†	<0.01
Smoking				0.001
Never	453 (59.5)	111 (56.4)	140 (46.5)	
Past or current smoker	249 (32.7)	70 (35.5)	141 (46.8)	
Unknown	60 (7.9)	16 (8.1)	20 (6.6)	
Alcohol intake				<0.01
None	494 (64.8)	134 (68.0)	150 (49.8)	
Moderate	268 (35.2)	63 (32.0)	151 (50.2)	
Systolic blood pressure (mmHg)	123.6 (18.0)	124.6 (16.8)	127.2 (17.6)*	0.01
Diastolic blood pressure (mmHg)	73.5 (10.5)	75.4 (10.7)	76.9 (10.2)*	<0.01
Fasting glucose (mg/dL)	95.0 (14.7)	97.8 (20.2)	110.5 (23.7)*†	<0.01
HDL cholesterol (mg/dL)	58.8 (15.1)	50.6 (13.2)*	50.8 (12.1)*	<0.01
Triglycerides (mg/dL)	89 (67–121)	132 (96–183)*	130 (95–176)*	<0.01
Total cholesterol (mg/dL)	193.2 (36.3)	198.6 (35.6)	189.7 (36.8)†	0.03
CSVD markers (≥1)	165 (21.6)	43 (21.8)	99 (32.9)	<0.01
Presence of moderate to severe WMH	73 (9.6)	19 (9.6)	60 (19.9)	<0.01
Presence of microbleeds	74 (9.71)	18 (9.1)	37 (12.3)	0.30
Presence of lacunes	64 (8.4)	12 (6.1)	37 (12.3)	0.041

Data are presented as mean (SD) or number (percentage).

*NAFLD* nonalcoholic fatty liver disease; *NFS* NAFLD fibrosis score, *BMI* body mass index, *HDL* high-density lipoprotein, *MMSE* Mini-Mental State Examination, *CSVD* cerebral small vessel disease, *WMHs* white matter hyperintensitie.

*P < 0.05 compared to no NAFLD; ^†^P < 0.05 compared to FIB-4 < 1.45.

### Association between NAFLD and CSVD markers

First, the crude odds ratio (OR) for moderate to severe WMH comparing participants with NAFLD to those without it was 1.78 (95% confidence interval (CI): 1.27–2.50). (Model 1). This association remained significant after adjusting for age, sex, smoking, alcohol, obesity, hypertension, diabetes, and hyperlipidemia (OR: 1.64; 95% CI: 1.10–2.42). However, the associations between NAFLD and the presence of lacunes and MBs were not significant, as crude ORs for lacunes and MB were 1.19 (95% CI: 0.81–1.76) and 1.15 (95% CI: 0.66–1.55), respectively.

When we assessed these associations according to the severity of NAFLD, the OR (95% CI) for moderate to severe WMH in participants with a low FIB-4 (<1.45) and with intermediate to high FIB-4 (≥1.45) were 1.14 (0.72–1.82) and 1.77 (1.13–2.78) compared to participants without NAFLD, respectively. Especially, the linear trend test showed a significant association between the severity of NAFLD fibrosis (non-NAFLD, NAFLD with FIB-4 < 1.45 or NAFLD with FIB-4 ≥1.45) and the presence of moderate to severe WMH (*p* for trend = 0.016) (Table 3).

**Table 3:** Adjusted odds ratios (95% CI) for presence of moderate to severe white matter hyperintensities, lacunes, and microbleeds by nonalcoholic fatty liver disease (NAFLD) severity (*n* = 1,260).

	None (*n* = 762)	NAFLD (*n* = 498)	*p* value	NAFLD with FIB-4 < 1.45 (*n* = 259)	NAFLD with FIB-4 ≥ 1.45 (*n* = 239)	*p* for trend
WMH						
Crude model	*reference*	1.78 (1.27, 2.50)	0.001	1.45 (0.92, 2.20)	2.19 (1.46, 3.28)	<0.001
Model 1	*reference*	2.07 (1.44, 2.96)	<0.001	1.45 (0.94, 2.23)	2.44 (1.61, 3.69)	<0.001
Model 2	*reference*	1.64 (1.10, 2.42)	0.014	1.14 (0.72, 1.82)	1.77 (1.13, 2.78)	0.016
Lacunes						
Crude model	*reference*	1.19 (0.81, 1.76)	0.38	0.86 (0.51, 1.47)	1.57 (0.99, 2.48)	0.11
Model 1	*reference*	1.19 (0.80, 1.77)	0.39	0.86 (0.50, 1.46)	1.48 (0.92, 2.36)	0.18
Model 2	*reference*	0.99 (0.93, 1.54)	0.95	0.69 (0.39, 1.22)	1.14 (0.68, 1.88)	0.79
Microbleeds						
Crude model	*reference*	1.15 (0.80, 1.67)	0.45	0.95 (0.59, 1.54)	1.39 (0.89, 2.17)	0.22
Model 1	*reference*	1.16 (0.80, 1.69)	0.43	0.95 (0.58, 1.53)	1.34 (0.85, 2.11)	0.28
Model 2	*reference*	1.01 (0.66, 1.55)	0.95	0.78 (0.47, 1.31)	1.11 (0.68, 1.81)	0.83

*FIB-4* Fibrosis-4 index*; *WMH* white matter hyperintensities.

Model 1: Adjusted for age and sex.

Model 2: Further adjusted for smoking (never vs. past or current smokers), alcohol consumption (none vs. moderate), obesity (not obese vs. obese), hypertension, diabetes, and hyperlipidemia.

*For the FIB-4, the model was not adjusted for age, as this factor is included in the calculation of the FIB-4.

When the analyses were conducted with NAFLD categories using NFS, the results were same as those seen with FIB-4 index, as the association between the presence of moderate to severe WMH and NAFLD with a low NFS (<−1.455) was not significant (OR: 0.92; 95% CI: 0.53–1.59), while its association with an intermediate to high NFS (≥−1.455) was significant (OR: 2.05; 95% CI: 1.34–3.14). A linear trend test showed a significant association between the severity of NAFLD fibrosis and the presence of moderate to severe WMH as well (*p* for trend = 0.002) (Table 4).

**Table 4:** Adjusted odds ratios (95% CI) for presence of moderate to severe white matter hyperintensity, lacunes, and microbleeds by non-alcoholic fatty liver disease (NAFLD) severity (*n* = 1,260).

	None (*n* = 762)	NAFLD (*n* = 498)	*p* value	NAFLD with NFS < −1.455 (*n* = 197)	NAFLD with NFS ≥ −1.455 (*n* = 301)	*p* for trend
WMH						
Crude model	*reference*	1.78 (1.27, 2.50)	0.001	1.01 (0.59, 1.71)	2.35 (1.62, 3.41)	<0.001
Model 1	*reference*	2.07 (1.44, 2.96)	<0.001	1.01 (0.59, 1.72)	2.61 (1.78, 3.82)	<0.001
Model 2	*reference*	1.64 (1.10, 2.42)	0.014	0.92 (0.53, 1.59)	2.05 (1.34, 3.14)	0.002
Lacunes						
Crude model	*reference*	1.19 (0.81, 1.76)	0.38	0.71 (0.37, 1.34)	1.53 (1.00, 2.35)	0.09
Model 1	*reference*	1.19 (0.80, 1.77)	0.39	0.71 (0.37, 1.34)	1.45 (0.94, 2.24)	0.15
Model 2	*reference*	0.99 (0.93, 1.54)	0.95	0.63 (0.33, 1.22)	1.20 (0.74, 1.95)	0.61
Microbleeds						
Crude model	*reference*	1.15 (0.80, 1.67)	0.45	0.93 (0.54, 1.61)	1.30 (0.86, 1.98)	0.26
Model 1	*reference*	1.16 (0.80, 1.69)	0.43	0.93 (0.54, 1.61)	1.27 (0.83, 1.94)	0.33
Model 2	*reference*	1.01 (0.66, 1.55)	0.95	0.86 (0.50, 1.49)	1.09 (0.68, 1.75)	0.80

*NFS* NAFLD (Non-Alcoholic Fatty Liver Disease) Fibrosis Score*, *WMH* white matter hyperintensities.

Model 1: Adjusted for age and sex.

Model 2: Further adjusted for smoking (never vs. past or current smokers), alcohol consumption (none vs. moderate), obesity (not obese vs. obese), hypertension, diabetes, and hyperlipidemia.

*For the NFS, the model was not adjusted for age and diabetes. Because that variable already included in the NFS formula.

**Figure 1:**
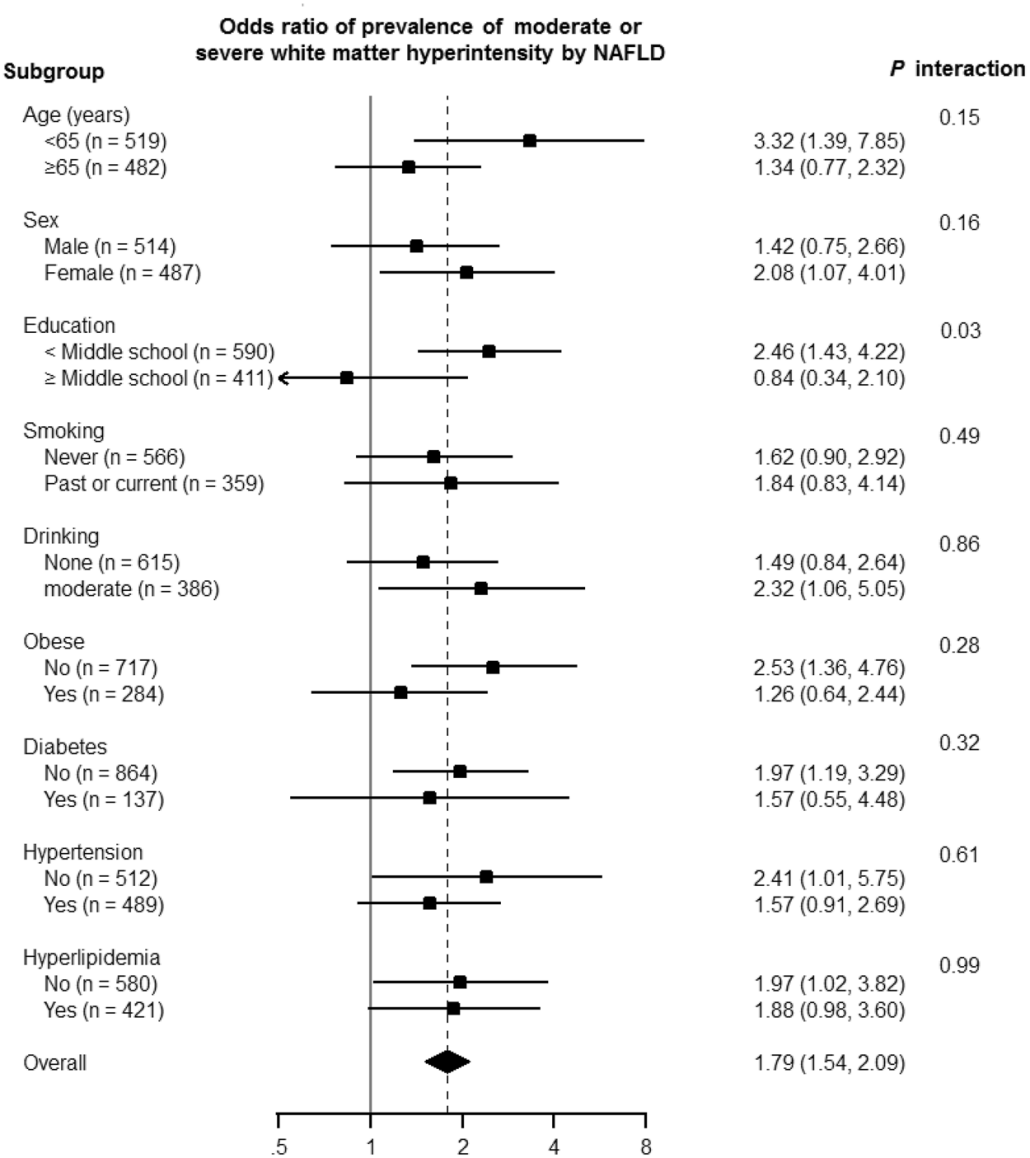
Odds ratios for prevalence of moderate or severe white matter hyperintensities by non-alcoholic fatty liver disease with FIB-4 ≥ 1.45 in predefined subgroups.

Additionally, we evaluated the association between NAFLD with FIB-4 ≥1.45 with the prevalence of moderate or severe WMH in different clinical subgroups (Fig. 1). The level of association between NAFLD with FIB-4 ≥1.45 and the presence of moderate to severe WMH was significantly different according to the educational level (*p* for interaction = 0.03).

## Discussion

This study determined that NAFLD has a significant association with the presence of WMHs, even after controlling for cardiometabolic risk factors. This study included a large number of participants (n = 1,260) and extensive data about comorbid cardiometabolic risk factors and various CSVD MRI markers, key issues in older patients. These strengths enabled us to examine the independent relationship between NAFLD and CSVD, regardless of various cardiometabolic risk factors. Therefore, our findings provide insight into the contribution of NAFLD to development of CSVD.

Our major finding was that NAFLD has a significant association only with WMH among different CSVD markers, even after controlling for cardiometabolic risk factors. Especially, when we divided patients into two groups according to fibrosis score, association with WMH was maintained only in NAFLD with an intermediate to severe fibrosis score, suggesting that the severity of NAFLD, rather than the presence of NAFLD itself, might be more importantly related to the development of CSVD. This finding is consistent with a recent study which reported that patients with NAFLD with fibrosis have a higher risk of having WMH than patients without NALFD or patients with NALFD without fibrosis^8^. However, our findings showed that there was an increase in the frequency of moderate to severe WMH with increasing fibrosis severity.

It is difficult to clarify the exact pathomechanism of the association between NAFLD and WMH. However, there are several explanations for this finding. First, NAFLD contributes to decreased cerebrovascular reactivity, causing chronic hypoperfusion and subsequent WMH. The pathogenesis of WMH has not been identified, but endothelial dysfunction could decrease cerebrovascular reactivity^9^ and chronic hypoperfusion. NAFLD is closely related to insulin resistance^10^ and metabolic risk factors, which is significantly associated with endothelial dysfunction^11^. Second, NAFLD could be related to subclinical inflammation, similar to other metabolic syndromes which contribute to blood brain barrier (BBB) disruption^12^, and progress to WMH^13^, despite the unknown relationship between systemic inflammation and CSVD. NAFLD with advanced fibrosis might contribute to systemic inflammation more than NAFLD without fibrosis. Finally, NAFLD is related to carotid atherosclerosis^14^, which might cause microembolic events, which could in turn lead to increased WMH. The aforementioned processes could also be interrelated, rather than occurring independently.

The level of association between NAFLD and the presence of moderate to severe WMH was significantly different according to the educational level of the patients (*p* for interaction = 0.03). This observation has led us to consider the reason for the effect of different educational levels on the NAFLD-WMH association. Interestingly, the lower educational group had a stronger association between NAFLD and WMH. One of possible explanations for this is that education is a representative measure of socioeconomic status (SES)^15^. Therefore, patients with a lower SES might have more health-related risk factors, including cardiovascular risk factors^16^ and other unmeasured confounding variables, such as dietary and lifestyle risk factors, which make patients in this group more vulnerable to developing WMH in response to the same metabolic stress.

In this study, NAFLD was not associated with the formation of lacunes or MBs. Unlike our findings, a recent study showed fatty liver disease is reported to be associated with lacunar infarct especially in non-obese population^17^. However, they included alcoholic fatty liver disease as well as NAFLD. WMH, lacunes, and MBs are CSVD MRI markers, but they have slightly different pathogeneses. WMH are related to chronic, diffuse, and subclinical ischemia, and BBB disruption^18,19^. Lacunes are attributed to acute, severe, or localized ischemia^18^, and MBs result from focal bleeding in small vessels that are either damaged by lipohyalinosis or by amyloid angiopathy^20^. Therefore, we can assume that NAFLD may lead to increased inflammation or cerebrovascular reactivity, and cause diffuse and chronic hypoperfusion or BBB disruption, rather than focal ischemia or microhemorrhages.

In this study, we investigated the relationship between CSVD and NAFLD in a large study with cognitively normal individuals. However, there were several limitations to this study. First, our study was cross-sectional, precluding claims of causality. The temporal relationship between NAFLD and CSVD markers remains unclear. Longitudinal studies are needed to determine the impact of NAFLD on the incidence of CSVD. Second, we defined NAFLD by US, which cannot detect mild steatosis and is operator-dependent. We also calculated the NAFLD FIB-4/NFS index to represent the severity of NAFLD, because simple US cannot differentiate steatohepatitis from simple steatosis^21^. In addition, while the accuracy of US for establishing the presence of fatty liver is high, it is subject to measurement error^22^. Finally, this study was conducted in Koreans who underwent health screening exams, and thus may not be generalizable to other settings or to other ethnicities.

In conclusion, our findings suggest that NAFLD might be a potential independent risk factor for the presence of moderate to severe WMH. Therefore, evaluation of conventional risk factors in patients with NAFLD warrants the presence of CSVD. This is of particular importance, given that there is an increasing prevalence of NAFLD, which can be potentially controlled by lifestyle modification, such as weight reduction, regular exercise, and changes in dietary patterns. Therefore, additional efforts to evaluate and manage NAFLD as one of several important cardiometabolic risk factors in patients with CSVD are needed.

## Methods

### Study participants

**Figure 2:**
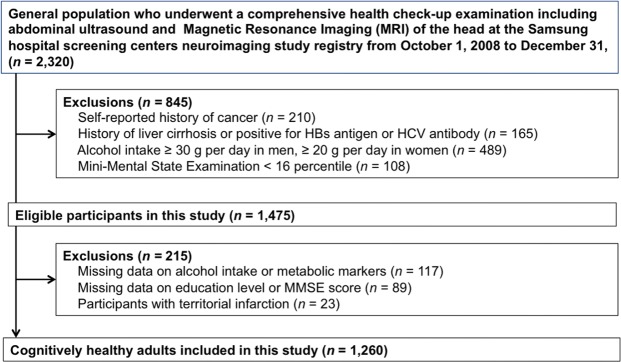
Flow chart of study participants.

The study population was comprised of men and women 40 years of age or older who underwent a health screening exam at the Health Promotion Center of the Samsung Medical Center in Seoul, Korea from October 1, 2008 to December 31, 2013. We included 2,320 subjects who had undergone at least one neurological and neurocognitive screening test, including brain MRI and the Mini-Mental Status Examination (MMSE), as well as an abdominal US. We excluded participants who had any of the following conditions: history of cancer (n = 210), history of liver cirrhosis/positive hepatitis B surface antigen/hepatitis C virus antibodies (n = 165), alcohol intake ≥30 g/day in men or ≥20 g/day in women (n = 489), or that scored below the 16^th^ percentile in age-, sex-, and education-matched norms according to the MMSE (n = 108). Patients that had structural lesions, including territorial cerebral infarctions, brain tumors, or intracranial hemorrhage on brain MRI were also excluded (n = 23). In addition, we excluded participants who had missing data on alcohol intake (n = 117), education, or MMSE score (n = 89), resulting in a final sample size of 1,260 (640 men and 620 women). (Fig. 2).

This study was approved by the Institutional Review Board of the Samsung Medical Center. The requirement for informed consent was waived because we only used de-identified data collected for clinical purposes during health screening exams.

### Data collection

To evaluate the presence of NAFLD, an abdominal US was performed using the LogiQ E9 (GE Healthcare, Milwaukee, WI, USA), iU22 xMatrix (Philips Medical Systems, Cleveland, OH, USA) or ACUSON Sequoia 512 (Siemens, Issaquah, WA, USA) US machine, and the procedure was performed by experienced radiologists blinded to the study aims. Images were captured in a standard fashion with the patients in the supine position with their right arm raised above their head. A US diagnosis of fatty liver was made based on standard criteria, including parenchymal brightness, liver-to-kidney contrast, deep beam attenuation, and bright vessel walls^23,24^. Because we excluded participants with excessive alcohol use (≥30 g/day for men and ≥20 g/day for women), as well as other identifiable causes of fatty liver at baseline, a US-diagnosed fatty liver was considered NAFLD. To assess the severity of fibrosis, we calculated Fibrosis-4 (FIB-4) index as (age (years) x AST (U/L))/(platelet (10^9^/L) x (ALT(U/L)^1/2^), and stratified NAFLD patients according to their FIB-4 index as having an intermediate to high (FIB-4 ≥1.45) or low (FIB-4 < 1.45) probability of advanced fibrosis^25^. A low FIB-4 (<1.45) is also a strong predictor of the absence of liver fibrosis. For sensitivity analysis, we also classified patients into two groups using NAFLD fibrosis score (NFS), which was calculated as −1.675 + 0.037 × age (years) + 0.094 × BMI (kg/m^2^) + 1.13 × impaired fasting glucose/diabetes (yes = 1, no = 0) + 0.99 × AST/ALT ratio − 0.013 × platelet count (×10^9^/l) − 0.66 × albumin (g/dl)^26^, with subjects having either a high-intermediate (NFS ≥ −1.455) or low (NFS < −1.455) probability of advanced fibrosis.

### Brain MRI

All participants underwent brain MRI, including T2* gradient echo (GRE) and three-dimensional (3D) Fluid-attenuated inversion recovery (FLAIR) imaging, using the same type of 3.0 T MRI scanner (Philips 3.0 T Achieva, Best, the Netherlands). The following parameters were used for the T2* GRE images: axial slice thickness, 5.0 mm; inter-slice thickness, 2 mm; repetition time (TR), 669 ms; echo time (TE) 16 ms; flip angle, 18°; matrix size, 560 × 560 pixels. 3D FLAIR images were acquired with the following imaging parameters: axial slice thickness, 2 mm; no gap; TR, 11,000 ms; TE, 125 ms; flip angle, 90°; and matrix size, 512 × 512 pixels.

### CSVD Markers on MRI

We used a modified Fazekas scale to visually rate WMH^27^. Periventricular WMH (PWMH) were classified as P1 (cap or band <5 mm), P2 (5 mm ≤ cap or band <10 mm), or P3 (cap or band ≥10 mm); deep WMH (DWMH) were classified into D1 (maximum diameter of deep white matter lesion <10 mm), D2 (10 mm ≤ lesion  <25 mm), or D3 (≥λ25 mm). The intra-class correlation coefficients for the inter-rater reliability of the WMH visual rating scale ranged from 0.73 and 0.91. PWMH and DWMH ratings were combined to give a final WMH classification of minimal (D1P1 or D1P2), moderate (D2P1, D3P1, D2P2, D3P2, D1P3, and D2P3), or severe (D3P3). In this study, the presence of WMH was determined when patients had moderate or severe WMH. This classification discriminates the presence of vascular risk factors and the severity of cerebrovascular disease markers^28^. Two experienced neurologists blinded to patient data reviewed images to determine the number and location of lacunes and MBs according to neuroimaging standards^29^. The kappa values for agreement between the two neurologists for the presence of lacunes and MBs were 0.78 and 0.92, respectively.

### Cardiometabolic risk factors

Serum total cholesterol, triglycerides, high-density lipoprotein (HDL) cholesterol, and low-density lipoprotein (LDL) cholesterol levels were determined using an enzymatic colorimetric method. Hyperlipidemia was defined according to the Adult Treatment Panel III^30^ criteria as triglyceride levels ≥150 mg/dl, HDL-cholesterol levels <40 mg/dl, or use of medication for dyslipidemia. Glucose was measured in blood samples that were collected after at least 10 hours of fasting. Diabetes was defined as a fasting serum glucose ≥126 mg/dL or self-reported use of insulin or antidiabetic medications. The Department of Laboratory Medicine and Genetics at the Samsung Medical Center has participated in several proficiency testing programs operated by the Korean Association of Quality Assurance for Clinical Laboratory, the Asian Network of Clinical Laboratory Standardization and Harmonization, and the College of American Pathologists.

Smoking status was categorized as never, past, or current smoker. Alcohol intake was categorized as either none or moderate (<30 g/day in men and <20 g/day in women). Trained nurses measured the height, weight, and sitting blood pressure of participants wearing a lightweight hospital gown and no shoes. Body mass index (BMI) was calculated as weight in kilograms divided by height in meters squared. BMI was classified according to Asian-specific criteria^31^, and obesity was defined as a BMI ≥25 kg/m^2^. Hypertension was defined as a systolic blood pressure ≥140 mm Hg, a diastolic blood pressure ≥90 mmHg, or current use of antihypertensive medications.

### Statistical analysis

An analysis of variance was used to compare the demographics and clinical characteristics of three groups: no NAFLD, NAFLD with a low FIB-4 (<1.45) and NAFLD with an intermediate to high FIB-4 (≥1.45). After the test, we conducted post hoc test using Scheffé's method. A multivariable logistic regression analysis was performed to assess the association between NAFLD and CSVD markers. We also conducted the same analysis for three groups categorized by NFS: no NAFLD, NAFLD with a low NFS (< −1.455) and NAFLD with an intermediate to high NFS (≥−1.455).

We used three models with increasing degrees of adjustment to account for potential confounding factors at baseline. To address missing data, we used a missing-indicator method to create a missing value category for each incomplete, independent, categorical variable^32^. The crude model was not adjusted for confounders. Model 1 was adjusted for age and sex. Model 2 was further adjusted for smoking (never vs. past or current smokers), alcohol consumption (none vs. moderate), obesity (not obese vs. obese), and metabolic factors, including hypertension, diabetes, and hyperlipidemia. Additionally, to test for linear trends according to NAFLD severity, we included the NAFLD severity category (no NAFLD, NAFLD with a low probability of fibrosis (FIB-4 < 1.45 or NFS < −1.455) and NAFLD with an intermediate to high probability of fibrosis (FIB-4 ≥ 1.45 or NFS ≥−1.455)) as a continuous variable in the logistic regression models.

In addition, we explored the association between NAFLD with FIB-4 ≥1.45 and the CSVD markers in pre-specified clinically relevant subgroups defined by age (<65 vs. ≥65 years), sex (women vs. men), education (<middle school vs. ≥middle school), smoking (never vs. past or current smokers), alcohol consumption (none vs. moderate), obesity (not obese vs. obese), hypertension (yes vs. no), diabetes (yes vs. no), and hyperlipidemia (yes vs.no). We tested for the interaction of NAFLD with clinical characteristics using Wald tests for cross-product terms in regression models. All reported p-values were two-sided, and the significance level was set at 0.05. All analyses were performed using STATA version 14 (StataCorp LP, College Station, TX, USA).
