# Author Correction: Non-alcoholic fatty liver disease and cerebral small vessel disease in Korean cognitively normal individuals

**DOI:** 10.1038/s41598-019-51401-8

**Published:** 2019-10-10

**Authors:** Hyemin Jang, Danbee Kang, Yoosoo Chang, Yeshin Kim, Jin San Lee, Ko Woon Kim, Young Kyoung Jang, Hee Jin Kim, Duk L. Na, Hee Young Shin, Mira Kang, Eliseo Guallar, Juhee Cho, Sang Won Seo

**Affiliations:** 1 Department of Neurology, Samsung Medical Center, Sungkyunkwan University School of Medicine, Seoul, 06351 Korea; 2 Neuroscience Center, Samsung Medical Center, 06351 Seoul, Korea; 3 Center for Clinical Epidemiology, Samsung Medical Center, 06351 Seoul, Korea; 4 Health Promotion Center, Samsung Medical Center, 06351 Seoul, Korea; 5 Department of Occupational and Environmental Medicine, Kangbuk Samsung Hospital, Sungkyunkwan University, School of Medicine, Seoul, Korea; 6 Department of Neurology, Kyung Hee University Hospital, Seoul, Korea; 7 Department of Neurology, Chonbuk National University Hospital, Chonbuk National University Medical School, Jeonju, Korea; 8 Department of Health Sciences and Technology, Sungkyunkwan University, Seoul, 06351 Korea; 9 Clinical Research Design and Evaluation, SAIHST, Sungkyunkwan University, Seoul, 06351 Korea; 10 Department of Epidemiology, Johns Hopkins Medical Institutions, Baltimore, USA; 11 Department of Medicine, Johns Hopkins Medical Institutions, Baltimore, USA; 12 Welch Center for Prevention, Epidemiology and Clinical Research, Johns Hopkins Medical Institutions, Baltimore, USA

Correction to: *Scientific Reports* 10.1038/s41598-018-38357-x, published online 12 February 2019

The Acknowledgements section of this Article is incomplete.

“This work was supported by the National Research Foundation of Korea (NRF) grant funded by the Korea government (MSIP) (NRF-2017R1A2B2005081) and the Brain Research Program through the National Research Foundation of Korea (NRF) funded by the Ministry of Science, ICT & Future Planning (2016M3C7A1913844).”

should read:

“This work was supported by the National Research Foundation of Korea (NRF) grant funded by the Korea government (MSIP) (NRF-2017R1A2B2005081), the Brain Research Program through the National Research Foundation of Korea (NRF) funded by the Ministry of Science, ICT & Future Planning (2016M3C7A1913844), and a fund (2018-ER6203-00) by Research of Korea Centers for Disease Control and Prevention.”

